# Evaluation of Eating Habits and Their Impact on Health among Adolescents and Young Adults: A Cross-Sectional Study

**DOI:** 10.3390/ijerph18083996

**Published:** 2021-04-10

**Authors:** Sylwia Mizia, Anna Felińczak, Dariusz Włodarek, Magdalena Syrkiewicz-Świtała

**Affiliations:** 1 Department of Organisation and Management, Faculty of Health Sciences, Wroclaw Medical University, 51-618 Wroclaw, Poland; sylwia.mizia@umed.wroc.pl; 2 Department of Dietetics, Institute of Human Nutrition Sciences, Warsaw University of Life Sciences, 07-776 Warsaw, Poland; dariusz_wlodarek@sggw.edu.pl; 3 Department of Health Economics and Management, School of Public Health, Medical University of Silesia in Katowice, 41-902 Bytom, Poland; mswitala@sum.edu.pl

**Keywords:** health behaviours, pro-health diet index, obesity, overweight, high school students

## Abstract

According to the health field concept, the most important factor affecting health is a lifestyle. The current upward trend in overweight and obesity among younger populations is a consequence of inadequate lifestyle habits. The study aimed to characterise youth nutrition behaviour and knowledge in the context of the risk of developing overweight or obesity. The study group consisted of 307 high school students, 59% females and 41% males, aged between 15 and 19. Nutrition behaviours were studied using the standardised Questionnaire of Eating Behaviour. Body weight and body height were measured with a body composition analyser and a body height meter, respectively. It was observed that the average body mass index was 21.7 ± 3.4 kg/m^2^ for the females and 22.3 ± 3.1 kg/m^2^ for the males (*p* = 0.036). Disturbed weight-to-height ratios (i.e., overweight and obesity) were found in 15.6% of the females and 16.5% of the males. The diets of approximately 90% of these youth were characterised by excessively low pro-health product content. The males showed a significantly higher intensity of adverse health traits compared to the females (8.1% vs. 0.7%, *p* = 0.002). More than half of the males presented insufficient knowledge about food and nutrition (53.5% vs. 30.8%, *p* < 0.001). Regardless of gender, the study showed a positive correlation between adolescents’ level of knowledge and the pro-health diet index (gamma coefficient: 0.42, *p* < 0.001) and a negative correlation between their level of knowledge and the unhealthy diet index (gamma coefficient: −0.66, *p* < 0.001). The level of knowledge was closely related to the indicators of the intensities and adverse health characteristics of their diets. These results indicate the need for educational programs to raise awareness among youth in civilisation backgrounds.

## 1. Introduction

According to Lalonde’s health field concept, the most important factor influencing health is a lifestyle [1]. Inadequate lifestyle habits have led to a rise in overweight and obesity in young populations. According to the World Health Organization (WHO), children should be diagnosed as overweight and obese when their body mass index (BMI) is greater than or equal to the 85th percentile and the 95th percentile, respectively. For adults, the corresponding breakpoints are 25 kg/m^2^ and 30 kg/m^2^ [2].

Excess weight in the young population has serious health implications. The frequencies of endocrine, metabolic, orthopaedic and psychological disorders are significantly higher in children and adolescents with obesity than in peers with normal body weight. Research indicates that approximately 80% of obese adolescents will remain obese in adulthood [3].

Adolescence is a period of many essential changes in physical, psychological and social growth. It is also a key period in developing lifestyle and nutritional behaviours that may have various health implications for young individuals. One of the lifestyle components that are crucial for adolescent growth is a well-balanced diet [4].

Many authors highlight the insufficient knowledge of a healthy lifestyle among the young generation, including their choices regarding eating [5,6]. The most common nutrition mistakes committed by adolescents are eating meals irregularly, including omitting breakfast [4], consuming an improperly balanced diet and consuming large amounts of highly processed products and sweetened beverages [7,8,9].

In 2019, the 4th National Nutrition Conference was held to discuss why the obesity epidemic continuously grows despite the actions taken in 2006 at the WHO European Ministerial Conference on Counteracting Obesity in Istanbul. At that time, all European countries committed to implementing the strong, comprehensive actions recommended in the European Charter on Counteracting Obesity to achieve a reversal of the trend of increasing obesity by 2015 [10,11].

The “National Programme for the Prevention of Overweight and Obesity” was a response to the ideas contained in the Charter, signed by the Polish Minister of Health in 2006 [12]. One of the two specific objectives of the Program included preventing overweight and obesity, improving nutrition quality and physical activity of children and adolescents. As studies have shown, there has been no success in reversing and even stopping the trend of increasing obesity [13,14]. This problem is very complex and challenging to explain.

Knowledge of nutrition behaviours is an essential element in designing educational activities aimed at eliminating these undesirable habits. These actions may contribute to the reduction of excessive body weight among children and adolescents [15]. Research emphasises the effectiveness of programs promoting a healthy diet and physical activity and the importance of more personalised actions [16,17,18], as well as the need to identify potential barriers for such actions [19].

The primary purpose of this study was to characterise nutrition behaviours of adolescents and their knowledge about food and nutrition in the context of the risk of developing overweightness or obesity. The secondary aim was to identify improper dietary behaviours of adolescents, verify the relationship of these behaviours with their level of nutrition knowledge and indicate directions for educational activities aimed at increasing their awareness about the diseases of civilisation.

## 2. Materials and Methods

### 2.1. Participants and Settings

The study group consisted of 307 high school students in Wroclaw, Poland, 59% being females and the remainder (41%) being males. The participants were aged between 15 and 19 years and participated in a project titled “Assessment of high school students’ lifestyle”. This project was carried out by the Wroclaw Medical University, Poland, between 2016 and 2019. It studied participants’ nutrition and health status by performing basic anthropometric measurements and evaluated their knowledge about food and nutrition, eating habits and health behaviours using standardised questionnaires. A convenience sample was used based on who was willing to participate. Adult students who gave written consent to participate in the project and underage students whose parents or guardians gave written consent for their child to participate in the project were included in the study. In our opinion, the success rate should be more optimistic.

### 2.2. Sample Size

According to the City Office data in 2018, there were 10,354 high school students in Wroclaw. The minimum sample size was determined based on available literature [20,21,22], indicating that overweight or obesity affects approximately 20% of Polish children and adolescents. Assuming a margin of error of 5%, a confidence interval of 95% and 10% contingency for non-response were used to calculate a sample size of 240 students. The Central Statistical Office report on “Education in the 2017/2018 school year” [23] provides that the representation of women in high schools in the Lower Silesian Voivodship was equal to 60.8%, which was also reflected in the gender structure of our study group.

### 2.3. Ethical Considerations

The adult students gave written consent for their participation in the project, while the same was provided by parents or guardians of the underage students. The study was voluntary and anonymous. The students could withdraw from participation at any stage. The project was approved by the Bioethics Committee at the Wroclaw Medical University, Poland (KB–575/2016).

### 2.4. Study Outcomes

For the purposes of this study, the Questionnaire of Eating Behaviour (QEB) was used to assess the participants’ eating behaviours and opinions on food and nutrition [24,25]. The questionnaire covered an examination of: (i) Eating Habits (21 questions), (ii) Frequency of Food Consumption (21 questions), (iii) Opinion on Food and Nutrition (25 questions), (iv) Self-Assessment of Diet, Nutrition Knowledge and its Sources (4 questions), (v) personal data (8 questions). More detailed, the first and second parts of the questionnaire examined, the frequency of meal consumption and typical compositions of the participants’ meals (breakfast—12 items, dinner—14 items, supper—12 items), the frequency of selected product consumption (the scale: never/1–3 times a month/once a week/a few times a week/once a day/several times a day). The third part of the questionnaire consisted of 25 statements regarding food and nutrition, allowing the authors to assess the level of participants’ nutrition knowledge. During data processing, 37 respondents were removed from the data set for one of the following reasons: not answering the verification question (5 cases), not answering the question concerning the calculation of the diet indexes (31 cases) and not providing adequate answers (1 case).

Each participants’ body height was measured with a Seca 213 (Seca, Hamburg, Germany) body height meter (accuracy: 1 mm) while standing in an upright position without shoes. Each participant’s body weight was measured with a Tanita SC-240 MA (Tanita, Poznan, Poland) body composition analyser (accuracy: 100 g). The participant was requested to strip down to their underwear after emptying their bladder before the measurement was recorded.

BMI percentile grids for gender and age, developed as part of the OLAF and OLA project, were used to assess body mass levels, namely the degree of deficiency or excess of body mass, in the study population [26]. The remainder of the participants were classified according to the standards established by the WHO [27].

### 2.5. Statistical Analysis

Statistical analysis of the collected data was carried out using the statistical package STATISTICA version 12 (Tibco, Tulsa, OK, USA). Quantitative variables were depicted in terms of the mean value ± standard deviation as well as minimum and maximum. Qualitative variables were depicted in terms of percentage distribution. The Mann–Whitney U test and Chi-square test (with Yates’ correction where necessary) were used to compare the distributions of the quantitative and qualitative variables among the females and males, respectively. The choice of non-parametric methods was dictated by the failure to meet the assumption of the normal distribution of the measured variables verified with the Shapiro–Wilk test. The analysis of the relationships between the qualitative features measurable on an ordinal scale was carried out using the gamma coefficient. Results with *p* ≤ 0.05 were considered statistically significant.

## 3. Results

At the time of joining the project, the average age of the participants was 17.8 ± 0.9 years, with 15-year-olds accounting for less than 1%; 16-year-olds, for 15%; 17-year-olds, for 7%; 18-year-olds, for 61%; and 19-year-olds, for 16%. More than half of the students lived in a town with more than 100,000 residents and 28% resided in the countryside. Most of the participants (76%) lived with their parents and 17% resided with a multi-generational family. Less than 5% lived alone. Approximately 55% of the students assessed their financial situation as “average” and 37% as “above average”.

The average body height was 165.9 ± 6.0 cm for the females and 179.4 ± 6.4 cm for the males (*p* < 0.001), while the corresponding values for body weight were 59.8 ± 10.1 kg and 71.9 ± 11.8 kg (*p* < 0.001) (Table 1). The results of the analysis showed that the average BMI was 21.7 ± 3.4 kg/m^2^ for the females and 22.3 ± 3.1 kg/m^2^ for the males (*p* = 0.036), with 71.1% of the females and 78.0% of the males being within the norm (*p* = 0.170). Impaired weight–height proportions (i.e., overweight and obesity) were found in 15.6% of the females and 16.5% of the males (Table 1).

### 3.1. Frequency of Food Consumption

The diets of the males exhibited a significantly higher intensity of adverse health traits compared to those of the females (8.1% vs. 0.7%, *p* = 0.002), but they did not differ in terms of the intensity of the beneficial health features (Table 1).

Compared to the females, the males tended to consume fast food (23.4% vs. 7.8%, *p* < 0.001), fried food (73.0% vs. 54.5%, *p* = 0.002), cheese (73.0% vs. 57.8%, *p* = 0.011), meat products and meals (92.8% vs. 73.2%, *p* < 0.001), sweetened carbonated beverages (35.1% vs. 20.1%, *p* = 0.006) and alcoholic beverages daily (2.7% vs. 0%, *p* = 0.040) significantly more often (Table 2).

Moreover, compared to their female counterparts, the males consumed more often sweetened hot drinks with two or more teaspoons of sugar (45.0% vs. 29.6%, *p* = 0.011) and chose to drink strong alcoholic liquors (25.6% vs. 12.5%, *p* = 0.020) (Appendix A).

Further, the males consumed fruits significantly less frequently than the females (34.2% vs. 51.3%, *p* = 0.006), replacing them with fruit, vegetable, or fruit–vegetable juices (24.3% vs. 11.0%, *p* = 0.004) (Table 2).

### 3.2. Eating Habits

The analysis of eating habits showed that the students usually consumed 3–4 meals a day (Appendix A). The males ate meals at fixed times more often than the females (*p* = 0.012). Most of the students (approximately 83%) ate between meals, usually from several times a week to several times a day. The most frequently chosen snacks were fruits, which were significantly more often preferred by the females (61.5% vs. 47.1%, *p* = 0.041), as well as candies and cookies. Moreover, the females ate nuts, almonds, seeds and pips significantly more often (25.6% vs. 13.8%, *p* = 0.039).

More than 70% of the respondents reported typically consuming bread for breakfast, with mixed bread (wheat-rye) being the most popular choice, although the females ate wholemeal and Graham bread significantly more often than the males (21.4% vs. 11.5%, *p* = 0.043). Over 60% of the participants reported consuming butter and meat and ham; the latter was chosen significantly more often by the males (66.4% vs. 40.5%, *p* < 0.001). The females chose fruits or vegetables more often for breakfast (45.8% vs. 23.4%, *p* < 0.001). Most of the individuals consumed their first meal of the day with tea or coffee. Supper comprised items similar to those eaten at breakfast, although the males preferred hot meals (38.3% vs. 22.7%, *p* = 0.007). Approximately 65% of the students consumed soup during lunch. The second course in most cases consisted of meat, significantly more often for the males (94.6% vs. 75.3%, *p* < 0.001). The second course also included potatoes (consumed by >70% of the respondents) and grain products (eaten by 56% of the respondents), mostly rice and pasta, together with vegetable salads or cooked vegetables, especially among the females (57.8% vs. 41.4%, *p* = 0.009). The most commonly consumed meat was poultry and significantly more females than males reported preferring it (88.2% vs. 66.3%, *p* < 0.001). The males reported significantly more beef consumption (42.6% vs. 17.8%, *p* = 0.001), usually fried in vegetable oil or roasted. Most students did not add salt or added it only occasionally to the ready meals at the table. More than 70% of the students consumed plain water, with almost 40% reporting drinking at least six glasses a day.

### 3.3. Opinion on Food and Nutrition

Over half of the males were characterised as having an insufficient level of knowledge about food and nutrition (53.5% vs. 30.8%, *p* < 0.001) (Table 3). The analysis of the answers provided for the 25 questions on food and nutrition showed that more than half of the correct answers were given by 27% of the females and 15% of the males (*p* = 0.018). Moreover, 25% of the females and 46% of the males were unsure about their knowledge (i.e., they answered “I have no opinion” for more than half of the statements) (*p* < 0.001). The majority of the incorrect answers (>12) were provided by 2% of the males (none of the female respondents provided more than 12 incorrect answers).

Regardless of the gender, the study revealed a statistically significant positive correlation between the level of knowledge and the healthy diet index (gamma correlation coefficient: 0.42, *p* < 0.001) and a negative relationship between the level of nutrition knowledge and the unhealthy diet index (gamma correlation coefficient: −0.66, *p* < 0.001). Moreover, those who reported their financial situation as “above average” were significantly more likely to possess a good level of knowledge about food and nutrition (13.5% vs. 4.48%, *p* = 0.030).

### 3.4. Self-Assessment of Diet, Nutrition Knowledge and Its Sources

Nearly half of the students, regardless of gender, estimated that their diet on weekdays only slightly differs from that on weekends and holidays (Table 3). Similarly, gender played no role in evaluating the participant’s diet; the majority rated their diet as “good”. However, it is important to note that almost 40% of the participants assessed their diet as either “bad” or “very bad”. The female participants’ self-assessment of their nutrition knowledge was primarily “sufficient” (56%) or “good” (29%), while that of the males was “sufficient” (53%) but showed a clear shift in responses towards “insufficient” (21%). Approximately 50% of both genders indicated family and/or relatives as their most important source of nutrition knowledge. In addition, the responses indicated other sources of knowledge, namely websites, which were used more often by the females (56.9% vs. 45.5%, *p* = 0.079), as well as school and doctor, dietician or nutrition advisor, which were indicated equally by both genders.

## 4. Discussion

The increase in the number of young people being diagnosed as overweight or obese presents a major clinical problem. Worldwide, the number of overweight and obese children and adolescents (ages: 5–19 years) has increased tenfold in the last four decades [28]. In the last 20 years, the number of such cases has tripled in Poland. According to the available reports, overweight and obesity affect approximately 10% of young children (1–3 years), 30% of early school-age children and almost 22% of adolescents (those up to 15 years of age) in Poland [29].

The National Health and Nutrition Examination Survey (NHANES) data indicate that between 2015 and 2016, the occurrence of obesity was higher among adolescents aged 6–11 years (18.4%) and those aged 12–19 years (20.6%) compared to children aged 2–5 years (13.9%) [30]. According to the research conducted by the Food and Nutrition Institute in Warsaw (Poland), obesity affects approximately 16% of school children and adolescents (i.e., those aged 6–19 years), of which almost 15.4% are girls and 19.9% are boys [31]. A meta-analysis on the occurrence of obesity among Turkish children and adolescents revealed an increase from 0.6% to 7.3% between 1990–1995 and 2011–2015 [32]. Our study also shows excess body weight in 15.6% of the females and 16.5% of the males aged between 15 and 19 years.

Obesity in childhood and adolescence is associated with an increased likelihood of premature death and disability in adulthood, placing a heavy burden on individuals and societies worldwide while entailing enormous medical care and treatment costs and lowering the quality of life [33,34]. Nutrition behaviour, a lifestyle element, has a significant impact on health and quality of life. Poor nutrition behaviours may contribute to many disorders, such as diabetes, obesity, cardiovascular diseases, osteoporosis and posture defects [35].

Unfortunately, the results of many studies in Poland indicate an increase in nutrition irregularities, especially among children and adolescents. According to the recommendations concerning nutrition for young people, daily food intake should be divided into five meals at 3–4 h intervals. Our research results indicate that, on average, adolescents consumed 3–4 meals a day. Similar results were obtained by Czarniecka-Skubina and Namyslaw [36] for students aged 16–21 years. On the other hand, Kiciak et al. [37] found that half of the examined adolescents in their study ate 4–5 daily meals, with boys tending to eat more meals. These results were also confirmed by Kocka et al. [38], who surveyed a group of high school students and reported that more than half of the respondents (53.33%) consumed 4–5 meals a day. Interestingly, regular consumption of all meals was declared by only a small percentage of the participants (11.85%). It was also reported that almost thrice as high, but it is still lower than the ideal rate [38].

Consuming fewer meals during the day and taking longer breaks between them is conducive to snacking, another irregularity in eating habits. Over 80% of the youth in this research confirmed snacking between meals, from several times a week to several times a day. The most commonly chosen snacks were fruits and sweets. Similarly, Jasinska [18] reported that over 90% of the study participants admitted to snacking between meals with varying frequency, with boys tending to practice this habit more than girls and the most frequently declared snacks being sandwiches, fruit and fruit yoghurts. In addition, Wouters et al. [39] showed a significant relationship between the strength of the habit and the momentary energy intake from snacking between meals: the greater the strength of snacking between meals, the greater the amount of energy consumed.

More than half of the males and one-third of the females in this study, were characterised as having an insufficient level of knowledge about food and nutrition. This research confirms Cieslik et al.’s [40] findings, which unequivocally indicate that young people tend to have only a satisfactory nutrition knowledge level. A European multi-centre cross-sectional study called HELENA examined the nutrition knowledge of youth aged 12.5–17.5 years and confirmed low levels, indicating a positive relationship between nutrition knowledge and food choices [41]. Indeed, our research confirms these findings. This study also reported that the females had better nutrition knowledge, supported by research conducted at other centres. This result also corresponds with the common opinion that young females care more about their health and physical appearance than young males.

Given the low awareness of nutrition among youth, it is crucial to control their sources of nutrition-related knowledge. The results of this work indicate that adolescents typically obtain this information from their families. Parents influence their offspring through their views, dietary preferences, or behaviours. Frequently, insufficient parents’ nutrition knowledge may translate into dietary mistakes made by their children [42,43]. Thus, it is important to verify parents’ level of nutrition knowledge as well. However, this aspect was not considered during the design stage of this research, thus constituting a limitation of this study.

The available literature also emphasises the need to diversify youths’ diets with products from various food groups, given the need to supplement the body with all the nutrients required for health and growth. It is important to include vegetables and fruits in every meal because they are a rich source of antioxidants such as provitamin A, vitamin C, vitamin E and polyphenolic compounds, while avoiding potentially harmful products such as fast food. The frequency analysis of the consumption of various food groups revealed that the examined youth’s diet contained insufficient amounts of products with contents beneficial to health. This outcome may be due to the low variation in the youths’ diets, consequently exposing them to diseases resulting from nutrient deficiencies. Fortunately, potentially harmful food products rarely appeared in the diets of the respondents. The report “Health and Health Behaviours of Polish Residents in the Light of the European Health Interview Survey (EHIS) 2014” obtained from the Central Statistical Office showed that 73% of children consume fruits once a day or more often, while only 62% eat vegetables and 4% of children eat no vegetables or eat them less than once a week [44].

The increasing occurrence of overweight and obesity among children and adolescents requires dietary interventions to reverse this trend. This research indicates the necessity of monitoring and evaluating pro- and anti-health dietary behaviours, which will enable early diagnosis and prevention of diseases of civilisation. Focusing this process on the specific needs of a group will increase its effectiveness. The high percentage of young people with insufficient nutrition knowledge points to creating educational programs in this context. This study may be a starting point for such projects aimed at young people in Poland. The goal is to raise their level of nutrition literacy to encourage and consolidate positive health habits among them.

### Study Limitations

According to the authors, the main methodological limitation of the study was the use of the QEB questionnaire, which is a very extensive and detailed tool. The fact is that it allows a complete characterisation of eating behaviour and knowledge about food and nutrition. Nevertheless, the completion required focus and attention from the respondent, which can be difficult in this age group. According to the data processing procedure for the questionnaire, 12% of the respondents who answered the questions incorrectly or did not give all the answers were removed from the sample. This also may indicate that the QEB is too complicated an instrument to be used among young people, although according to the authors, it is dedicated from the age of 16. Further studies may also use more objective measurement tools to assess the impact of dietary habits on children and adolescents’ health status.

Another potential limitation may be the relatively small sample size (however, the estimation showed that 240 people is relevant for this study). This reflects the low level of interest among high school principals about participating in the project (25% positive responses). On the other hand, the coordinators appointed by the principals were able to encourage/recruit only 20–25% of given school students. The argument dominating the reasons for refusing to participate in the project was students’ need to complete compulsory educational activities, whereas a full survey required 60 min of commitment from a single respondent. Thus, further research needs to improve principals, teachers and students’ motivation to participate in the project.

## 5. Conclusions

The diet of the vast majority of the adolescents examined in this work was characterised by excessively low contents of pro-health food products. Every tenth young male was found to follow a diet that will likely increase their adverse health traits. Over half of the young males and every third young female in this study possessed insufficient knowledge about food and nutrition. Regardless of gender, the level of these youth’s knowledge was closely related to the intensities of beneficial and adverse health traits in a diet. Due to their insufficient knowledge, young people may make nutrition mistakes, resulting in the development of overweight and obesity and other diet-related diseases in the future. The irregularities identified in this study indicate the urgent need to educate young people about following pro-healthy eating habits and raise their awareness about the diseases of civilisation associated with poor eating habits.

### 5.1. Research Directions

It is important to prevent difficulties in recruiting participants for the project. For this purpose, it is necessary to establish cooperation with institutions that supervise public health in schools, making it mandatory for schools to participate in such a project. The study revealed that the time of measurements should be shortened, for instance, by creating two independent measuring stations. A platform for fulfilling the questionnaires via the internet should be created to avoid missing and incorrect data. The latter may include the options of individual results viewing and a simple statistical summary. In light of low nutritional awareness among young people, there is also a need to verify their parents’ nutritional knowledge, who constitute one of the primary sources of nutritional knowledge of students—their children.

### 5.2. Practical Implications

Although many national and regional programs on nutritional education and the realisation of nutritional content in the school curriculum, nutritional knowledge and the resulting behaviours in children and adolescents are still lacking. Therefore, educational activities should be integrated by introducing a school subject in nutrition, which teachers would introduce with appropriate professional qualifications and practical skills.

## Figures and Tables

**Table 1:** Baseline characteristics of study participants.

Variables		Sex		*p*-Value
		Female	Male	
Age (years)				
	M ± SD Min–Max	17.7 ± 0.9 16–19	17.9 ± 0.9 15–19	0.043
Height (cm)				
	M ± SD Min–Max	165.9 ± 6.0 149–181	179.4 ± 6.4 164–194	<0.001
Weight (kg)				
	M ± SD Min–Max	59.8 ± 10.1 38.8–101.6	71.9 ± 11.8 46.2–106.9	<0.001
BMI (kg/m2)				
	M ± SD Min–Max	21.7 ± 3.4 16.2–37.3	22.3 ± 3.1 16.7–33.1	0.036
Classification by BMI, *n* (%)				
	Underweight	24 (13.3%)	7 (5.5%)	0.170
	Normal	128 (71.1%)	99 (78.0%)	
	Overweight	24 (13.3%)	18 (14.2%)	
	Obesity	4 (2.2%)	3 (2.4%)	
Unhealthy diet index (point)				
	M ± SD Min–Max	11.6 ± 7.7 0.4–60.6	17.2 ± 11.0 2.1–54.0	<0.001
Unhealthy diet index, *n* (%)				
	Small	153 (99.4%)	102 (91.9%)	0.002
	Moderate	1 (0.7%)	9 (8.1%)	
	Big	0 (0%)	0 (0%)	
Pro-health diet index (point)				
	M ± SD Min–Max	21.6 ± 10.3 3.0–51.8	20.6 ± 11.5 3.8–75.0	0.212
Pro-health diet index, *n* (%)				
	Small	133 (86.4%)	99 (89.2%)	0.335
	Moderate	21 (13.6%)	11 (9.9%)	
	Big	0 (0%)	1 (0.9%)	

**Table 2:** Women and men’s food consumption frequency.

Variables		Sex		*p*-Value
		Female	Male	
Consumption of food included in the pro-health diet index at least once a week, *n* (%)				
	Wholemeal bread	73 (47.4%)	48 (43.2%)	0.502
	Milk	73 (47.4%)	60 (54.1%)	0.285
	Yoghurts	65 (42.2%)	47 (42.3%)	0.982
	Cottage cheese	48 (31.2%)	41 (36.9%)	0.327
	Fish	20 (13.0%)	13 (11.7%)	0.756
	Legume	25 (16.2%)	17 (15.3%)	0.840
	Fruits	141 (91.6%)	94 (84.7%)	0.081
	Fruits *	79 (51.3%)	38 (34.2%)	0.006
	Vegetables	142 (92.2%)	94 (84.7%)	0.053
Consumption of food included in the unhealthy diet index at least once a week, *n* (%)				
	Fast food	12 (7.8%)	26 (23.4%)	<0.001
	Fried food	84 (54.5%)	81 (73.0%)	0.002
	Cheese	89 (57.8%)	81 (73.0%)	0.011
	Sweets	104 (67.5%)	77 (69.4%)	0.751
	Sweets *	32 (20.8%)	33 (29.7%)	0.095
	Canned meat, fish and vegetables	5 (3.3%)	8 (7.2%)	0.141
	Sweetened carbonated drinks	31 (20.1%)	39 (35.1%)	0.006
	Energy drinks	16 (10.4%)	20 (18.0%)	0.074
	Alcoholic drinks	11 (7.1%)	15 (13.5%)	0.085
	Alcoholic drinks *	0 (0%)	3 (2.7%)	0.040
Consumption of the remaining products at least once a week, *n* (%)				
	Meat preserves and meals	112 (73.2%)	103 (92.8%)	<0.001
	Potatoes	85 (55.2%)	81 (73.0%)	0.003
	Powdered soups	11 (7.1%)	7 (6.3%)	0.789
	Fruit preserves	24 (15.6%)	10 (9.2%)	0.127
	Fruit, vegetable or fruit-vegetable juices	75 (48.7%)	65 (58.6%)	0.113
	Fruit, vegetable or fruit-vegetable juices *	17 (11.0%)	27 (24.3%)	0.004

* consumption at least once a day.

**Table 3:** Women and men’s level of nutritional knowledge, self-assessment of the diet and nutritional knowledge and its sources.

Variables		Sex		*p*-Value
		Female	Male	
The level of nutritional knowledge, *n* (%)				
	Insufficient	48 (30.8%)	61 (53.5%)	0.001
	Sufficient	95 (60.9%)	48 (42.1%)	
	Good	13 (8.3%)	5 (4.4%)	
Self-assessment of the diet on weekdays when compared to weekends and public holidays, *n* (%)				
	There is basically no difference	35 (22.7%)	29 (26.4%)	0.788
	It slightly differs	73 (47.4%)	49 (44.6%)	
	It differs significantly	46 (29.9%)	32 (29.1%)	
Self-assessment of the diet, *n* (%)				
supple	Very bad	3 (2.0%)	2 (1.8%)	0.930
	Bad	55 (35.7%)	36 (32.4%)	
	Good	87 (56.5%)	65 (58.6%)	
	Very good	9 (5.8%)	8 (7.2%)	
Self-assessment of nutritional knowledge, *n* (%)				
	Insufficient	16 (10.5%)	23 (20.7%)	0.112
	Sufficient	85 (55.6%)	59 (53.2%)	
	Good	44 (28.8%)	24 (21.6%)	
	Very good	8 (5.2%)	5 (4.5%)	
The most important sources of nutritional knowledge, *n* (%)				
	Family home and/or relatives	73 (50.7%)	52 (51.5%)	0.903
	Websites	82 (56.9%)	46 (45.5%)	0.079
	School	54 (37.5%)	33 (32.7%)	0.437
	Doctor, dietitian, nutritional advisor	35 (24.3%)	21 (20.8%)	0.519
	Daily press etc.	7 (4.9%)	3 (3.0%)	0.462
	Advertisement	4 (2.8%)	5 (5.0%)	0.374
	Radio and/or television	4 (2.8%)	5 (5.0%)	0.374

## Data Availability

The data presented in this study are available on request from the corresponding author (A.F.).

## Appendix A

**Table A1:** Women and men’s eating habits.

Variables		Sex		*p*-Value
		Female	Male	
The number of meals usually eaten during the day, n (%)				
1 meal		0 (0%)	2 (1.8%)	0.271
2 meals		18 (11.7%)	13 (11.7%)	
3 meals		48 (31.2%)	31 (27.9%)	
4 meals		70 (45.5%)	45 (40.5%)	
5 meals and more		18 (11.7%)	20 (18.0%)	
Meals eaten at regular times of the day, n (%)				
No		67 (43.5%)	45 (40.9%)	0.012
Yes, but only some		76 (49.4%)	44 (40.0%)	
Yes, all		11 (7.1%)	21 (19.1%)	
Snacking between meals, n (%)				
No		26 (16.9%)	18 (16.2%)	0.886
Yes		128 (83.1%)	93 (83.8%)	
The frequency of snacking between meals, n (%)				
Never		2 (1.5%)	1 (1.1%)	0.122
1-3 times in a month		9 (6.8%)	3 (3.2%)	
Once a week		15 (11.3%)	7 (7.4%)	
A few times a week		55 (41.4%)	32 (33.7%)	
Once a day		30 (22.6%)	22 (23.2%)	
A few times a day		22 (16.5%)	30 (31.6%)	
Food usually eaten between meals, n (%)				
Fruit		72 (61.5%)	41 (47.1%)	0.041
Vegetables		11 (9.4%)	7 (8.1%)	0.736
Yoghurts, cheese		34 (29.1%)	30 (34.5%)	0.409
Candies, cookies, cakes		43 (36.8%)	42 (48.3%)	0.099
Crackers, pretzels, chips		19 (16.2%)	23 (26.4%)	0.075
Nuts, almonds, seeds, pips		30 (25.6%)	12 (13.8%)	0.039
Products usually eaten for breakfast, n (%)				
Meat, ham		62 (40.5%)	71 (66.4%)	<0.001
Milk, yoghurts, kefirs		63 (41.2%)	45 (42.1%)	0.887
Cottage cheese, homogenized cheese		52 (34.0%)	26 (24.3%)	0.093
Cheeses		63 (41.2%)	55 (51.4%)	0.103
Eggs (in various forms)		66 (43.1%)	47 (43.9%)	0.9
Fish		4 (2.6%)	6 (5.6%)	0.217
Bread		112 (73.2%)	85 (79.4%)	0.248
Grain products		46 (30.1%)	36 (33.6%)	0.541
Jams, preserves, honey		33 (21.6%)	15 (14.0%)	0.123
Fruits or vegetables		70 (45.8%)	25 (23.4%)	<0.001
Fruit or vegetable juices		28 (18.3%)	15 (14.0%)	0.36
Tea, coffee		105 (68.6%)	61 (57.0%)	0.055
Products usually eaten at lunch, n (%)				
Soups		101 (65.6%)	73 (65.8%)	0.976
Meat		116 (75.3%)	105 (94.6%)	<0.001
Fish		36 (23.4%)	25 (22.5%)	0.871
Eggs (in various forms)		20 (13.0%)	22 (19.8%)	0.133
Milk, yoghurts, kefirs		8 (5.2%)	6 (5.4%)	0.94
Pancakes, croquettes, pies		42 (27.3%)	25 (22.5%)	0.38
Fruits		16 (10.4%)	20 (18.0%)	0.074
Vegetable salads		107 (69.5%)	65 (58.6%)	0.066
Boiled vegetables		89 (57.8%)	46 (41.4%)	0.009
Potatoes		108 (70.1%)	84 (75.7%)	0.319
Grain products		86 (55.8%)	62 (55.9%)	0.999
Bread		11 (7.1%)	17 (15.3%)	0.033
Desserts		11 (7.1%)	15 (13.5%)	0.085
Compotes, fruit or vegetable juices		31 (20.1%)	30 (27.0%)	0.188
Products usually eaten for dinner, n (%)				
Hot meals		34 (22.7%)	41 (38.3%)	0.007
Meat, ham		57 (38.0%)	70 (65.4%)	<0.001
Milk, yoghurts, kefirs		44 (29.3%)	42 (39.3%)	0.097
Cottage cheese, homogenized cheese		55 (36.7%)	27 (25.2%)	0.053
Cheese		55 (36.7%)	59 (55.1%)	0.003
Eggs (in various forms)		43 (28.7%)	39 (36.5%)	0.187
Fish		10 (6.7%)	9 (8.4%)	0.598
Bread		104 (69.3%)	81 (75.7%)	0.263
Jams, preserves, honey		30 (20.0%)	17 (15.9%)	0.401
Fruits or vegetables		67 (44.7%)	30 (28.0%)	0.007
Fruit or vegetable juices		18 (12.0%)	20 (18.7%)	0.136
Tea, coffee		91 (60.7%)	52 (48.6%)	0.055
A type of bread eaten most often, n (%)				
I don’t eat bread		4 (2.9%)	0 (0%)	0.028
Mixed		68 (48.6%)	51 (49.0%)	
Wheat		33 (23.6%)	39 (37.5%)	
Wholemeal, grahams		30 (21.4%)	12 (11.5%)	
Crispy, rice		5 (3.6%)	2 (1.9%)	
Grain products usually consumed, n (%)				
Oatmeal		33 (26.4%)	16 (16.2%)	0.066
Cereals		39 (31.2%)	35 (35.4%)	0.512
Semolina		2 (1.6%)	3 (3.0%)	0.472
Barley		6 (4.8%)	4 (4.0%)	0.785
Buckwheat		12 (9.6%)	10 (10.1%)	0.9
Rice		66 (52.8%)	48 (48.5%)	0.521
Pasta		72 (57.6%)	62 (62.6%)	0.446
Milk and dairy products consumed most often, n (%)				
With standard fat content		64 (45.4%)	72 (66.1%)	0.002
Low in fat		69 (48.9%)	36 (33.0%)	
Without fat		8 (5.7%)	1 (0.9%)	
A type of meat usually eaten, n (%)				
Pork		65 (48.2%)	60 (59.4%)	0.087
Beef		24 (17.8%)	43 (42.6%)	0.001
Veal		1 (0.7%)	1 (1.0%)	0.836
Mutton, lamb		1 (0.7%)	2 (2.0%)	0.4
Venison		3 (2.2%)	0 (0%)	0.132
Poultry		119 (88.2%)	67 (66.3%)	<0.001
A preparation method of consumed meat, n (%)				
Boiled		43 (32.8%)	36 (34.6%)	0.773
Stewed		24 (18.3%)	19 (18.3%)	0.909
Grilled		14 (10.7%)	7 (6.7%)	0.291
Baked		70 (53.4%)	43 (41.4%)	0.065
Fried		86 (65.7%)	74 (71.2%)	0.369
A number of servings of fruits and vegetables consumed throughout the day, n (%)				
I don’t eat them at all		2 (1.3%)	3 (2.7%)	0.012
1 portion		32 (20.8%)	44 (39.6%)	
2 portions		56 (36.4%)	34 (30.6%)	
3 portions		44 (28.6%)	17 (15.3%)	
portions		10 (6.5%)	5 (4.5%)	
5 portions and more		10 (6.4%)	8 (7.2%)	
The most frequently used frying fat, n (%)				
I don’t use any fat		8 (5.5%)	3 (2.8%)	0.767
I use different types of fat depending on the dish		54 (37.2%)	43 (40.2%)	
Vegetable oil		70 (48.3%)	50 (46.7%)	
Margarine		4 (2.8%)	2 (1.9%)	
Butter		9 (6.2%)	9 (8.4%)	
Lard		0 (0%)	0 (0%)	
The most frequently used spread fat, n (%)				
I don’t use any fat		36 (23.5%)	18 (16.7%)	0.194
I use different types of fat, depending on what I have		11 (7.2%)	5 (4.6%)	
Mayonnaise		3 (2.0%)	3 (2.8%)	
Margarine		5 (3.3%)	11 (10.2%)	
Butter		96 (62.8%)	69 (63.9%)	
Fat mix		2 (1.3%)	2 (1.9%)	
Sweetening hot drinks, n (%)				
No		71 (46.7%)	39 (35.8%)	0.038
Yes, I sweeten it with one teaspoon of sugar		36 (23.7%)	21 (19.3%)	
Yes, I sweeten it with two or more teaspoons of sugar		45 (29.6%)	49 (45.0%)	
Adding salt to ready meals at the table, n (%)				
No		77 (50.7%)	47 (42.3%)	0.358
Yes, but only sometimes		57 (37.5%)	51 (46.0%)	
Yes		18 (11.8%)	13 (11.7%)	
A number of glasses of water that are usually drunk during the day, n (%)				
I don’t drink water at all		2 (1.3%)	3 (2.7%)	0.22
1 glass		14 (9.1%)	2 (1.8%)	
2 glasses		20 (13.0%)	10 (9.1%)	
3 glasses		22 (14.3%)	16 (14.6%)	
4 glasses		26 (16.9%)	20 (18.2%)	
5 glasses		15 (9.7)	11 (10.0%)	
6 glasses and more		55 (35.7%)	48 (43.6%)	
Type of water, n (%)				
Flavoured		5 (3.3%)	6 (5.5%)	0.317
Still		120 (79.5%)	78 (71.6%)	
Sparkling		26 (17.2%)	25 (22.9%)	
Type of alcoholic drinks drunk most often, n (%)				
Beer		39 (34.8%)	37 (47.4%)	0.003
Wine		35 (31.3%)	11 (14.1%)	
Cocktails		24 (21.4%)	10 (12.8%)	
Strong spirits		14 (12.5%)	20 (25.6%)	
